# Distress factors of voice‐hearing in young people and social relating: Exploring a cognitive‐interpersonal voice‐hearing model

**DOI:** 10.1111/papt.12411

**Published:** 2022-06-30

**Authors:** Aikaterini Rammou, Clio Berry, David Fowler, Mark Hayward

**Affiliations:** ^1^ School of Psychology University of Sussex Brighton UK; ^2^ Research & Development Department Sussex Partnership NHS Foundation Trust Brighton UK; ^3^ Brighton and Sussex Medical School University of Sussex Brighton UK

**Keywords:** adolescent, auditory hallucinations, early intervention, psychotic experiences, voice‐hearing, youth mental health

## Abstract

**Objectives:**

Little is known about the factors that can maintain the distress related to voice‐hearing experiences in youth. Building upon understandings developed with adults, this study aimed to explore the associations between negative relating between hearer and voices, persecutory beliefs about voices and voice‐related distress in a clinical sample of adolescents. The study also aimed to investigate associations between relating to voices and wider patterns of social relating.

**Design:**

This was an observational, cross‐sectional, survey study.

**Methods:**

Thirty‐four young people (age 14–18 years) who were hearing voices completed measures about voices (characteristics, relating and beliefs) and relating to social others (negative relating styles, social connectedness and belongingness). Participants were patients of NHS mental health services. Bivariate correlations explored associations between relating to voices and distress, beliefs about voices and distress, and between relating to voices and social relating variables.

**Results:**

Perceiving the voices as dominant, intrusive, and persecutory and resisting them was significantly associated with distress. Adjusting for loudness and negative content rendered the association between persecutory beliefs and distress non‐significant. Fear of separation and of being alone in relation to social others was associated with distancing from voices. Being suspicious, uncommunicative and self‐reliant and/or being sadistic and intimidating towards social others was significantly associated with dependence towards the voices. Greater hearer‐to‐voice dependence was associated with lower perceived social belongingness and connectedness.

**Conclusions:**

Beliefs about voices being persecutory, dominant, intrusive and resisting voices seem to be significant contributors of distress in young people. In terms of proximity and power, relating to voices and social others appears to be contrasting.

**Practitioner Points**

- Voice‐hearing in youth should be followed by a detailed assessment of young people’s experience, especially of factors that might be linked with distress, such as beliefs about voices, voice content and loudness as well as responses to voices.
- Voice loudness and negative content seem to be important distress contributors and could be markers for holding stronger beliefs about the persecutory nature of voices in young people with voice‐hearing experiences.
- Particular attention should be paid to young people who might seem dependent on their relationship with their voices as this might be reflecting difficulties in their social life.
- Psychological interventions targeting beliefs about voices, relating to voices and maladaptive responses to voices might be beneficial in reducing voice‐related distress in young people.

## INTRODUCTION

Auditory verbal hallucinations or voice‐hearing commonly refers to perceptual experiences of hearing a voice or voices in the absence of an appropriate external stimulus. Voice‐hearing takes place in full consciousness and is not voluntarily invoked (Slade & Bentall, 1988). Voice‐hearing is common amongst young people, in both clinical and non‐clinical populations (de Leede‐Smith & Barkus, 2013; Kelleher et al., 2014; Kelleher, Connor, et al., 2012; Kelleher, Keeley, et al., 2012). A recent meta‐analysis indicated a mean lifetime prevalence of 12.7% for children (5–12 years) and 12.4% for adolescents (13–17 years) (Maijer et al., 2018). Although for most young people voice‐hearing appears a transient phenomenon that will spontaneously resolve (Linscott & Van Os, 2013), this experience can be distressing and is linked to mental health problems such as depression, anxiety (Bartels‐Velthuis et al., 2016; De Loore et al., 2011; Jeppesen et al., 2015; Kelleher, Connor, et al., 2012; Kelleher, Keeley, et al., 2012; Ulloa et al., 2000) and psychotic disorders (Kelleher et al., 2014; Maijer et al., 2017; Sikich, 2013).

Although voice‐hearing in youth is relatively common (Maijer et al., 2018), very little is known about its phenomenology. In a sample of help‐seeking adolescents, Maijer et al. (2017) found that the majority of voice‐hearing were making comments or giving dangerous commands and were mostly not familiar voices. Most commonly, the voices had a normal speaking volume, they were experienced inside the young person's head followed by a mixed experience of hearing voices both inside and outside their head, in most cases taking place at random moments during their day, and the majority experiencing only negative voices. In a study with non‐help‐seeking adolescents, Coughlan et al. (2020) demonstrated that the richness and diversity of the phenomenology of voice‐hearing in young people is similar to adult samples (McCarthy‐Jones et al., 2014; Woods et al., 2015), ranging from once‐off experiences of low‐level benign mumbling to repeated experiences of clear voices, speaking in full sentences or conversing. The phenomenological diversity of young people's voice‐hearing has been further corroborated by a web‐based survey with adolescents (Parry & Varese, 2020) in which there was a distinction between pleasant and distressing voices. Pleasant voices seem to be discussed as having human qualities such as motivations, emotions and gender, whereas negative and distressing voices seemed to be described as ‘ghosts’ or ‘whispers’ with commanding or threatening content (Parry & Varese, 2020).

Psychological models have suggested why hearing voices can be distressing for adults, identifying the processes that can elicit and/or maintain distress in order to inform psychosocial interventions. Cognitive models postulate that the hearers' beliefs about voices, especially intent (e.g. malevolence), power (e.g. omnipotence) and social rank (e.g. powerlessness and subordination to voices) (e.g. Birchwood et al., 2000, 2004; Birchwood & Chadwick, 1997) are key contributors to voice‐related distress, irrespective of the phenomenological characteristics of voices (e.g. frequency, loudness) or voice content (Birchwood & Chadwick, 1997; Chadwick & Birchwood, 1994, 1995). Adding an interpersonal component to the cognitive model of voice‐hearing, further research has indicated that the way people relate to voices (e.g. Hayward, 2003), such as relating from a position of distance to the voices and the voices being perceived as relating intrusively and dominantly to the hearer (Hayward et al., 2008; Sorrell et al., 2010; Vaughan & Fowler, 2004), may also be associated with voice‐related distress.

Although seemingly independent from the impact of beliefs about voices (Peters et al., 2012; Van der Gaag et al., 2003), voice content is associated with patient status or a need for care for people with this experience (Daalman et al., 2011; Johns et al., 2014; Kråkvik et al., 2015). Recent research has also highlighted the role of negative voice content in voice‐related distress, emphasising a potential causal pathway mainly from adverse life experiences to voice‐related outcomes (Larøi et al., 2019).

To further the understanding of the voice‐hearing experience within an interpersonal framework, an evolving literature has explored the similarities of relating to voices and social others (Hayward et al., 2011). Birchwood et al. (2000) found that power and social rank differentials between clinical groups of hearers and others in social relationships were similar to the ones between the individuals and their voices. Complementing these findings by focusing on both the proximity (distance/closeness) and power (upperness or dominance/lowerness or dependence) in the hearer‐voice relationship, Hayward (2003) showed that the relationships between patients and their voice had similarities to social relating, specifically with regard to dominant, dependent and closeness styles of relating, while controlling for beliefs about voices and emotional distress. Hayward (2003) also found that relating from a position of distance was unique to relating with voices, especially when voices had no perceived identity for the hearer, or when voices were perceived as dominant (Hayward et al., 2008). Adding to this, a systematic review has further supported that voice‐hearers who perceive themselves to be of low social rank relative to others also feel inferior in relation to their voice and respond accordingly (Paulik, 2012).

To date, the fit of cognitive model of voice‐hearing (Birchwood & Chadwick, 1997) has only been tested to young people in one clinical study (Cavelti et al., 2019, 2020). In a clinical sample of 15‐ to 25‐year‐olds, Cavelti et al. (2019) found that beliefs about voice malevolence, omnipotence and high social rank, as well as negative voice content, were associated with general distress (depression and anxiety) and that negative beliefs about voices explained variance in depression over and above negative voice content. Consistent with adult findings, beliefs about malevolent voice intent were correlated with more resistance towards voices, and beliefs about benevolence with more engagement with voices. Using the same sample, Cavelti et al. (2020) found that both beliefs about voice omnipotence and malevolence, and negative beliefs about self and social others, were important determinants of depression in youth with voice‐hearing experiences.

Two recent studies have additionally provided support for adopting a relational account of distress in youth with voice‐hearing. By using a nuanced first‐person perspective analysis of online survey data, Parry and Varese (2020) reported that distressing voices were mostly described with a sense of relational distance, which is consistent with adult literature (Hayward, 2003). Moreover, findings indicated that voices often reflected how young people experienced close social relationships, potentially showing an important role for the influence of appraisals of social others on young people's voice‐hearing experience and/or vice versa (Parry et al., 2020).

Considering that adolescence is described as a life period characterised by changes in how young people perceive, understand and interact with social others (Blakemore, 2008; Pachucki et al., 2015), further investigation of how relating to voices might impact on young people's social relationships, and vice versa, is warranted. Adult research has suggested that hearers could spend a substantial amount of time talking to voices, creating interpersonal relationships with them (Corstens et al., 2012) and that voices could be fulfilling social needs that are not met in other relationships (Beavan & Read, 2010; Corstens et al., 2012; Mawson et al., 2010, 2011), potentially leading to spending less time in other social interactions (Favrod et al., 2004). Parry et al. (2020) reported that young people commonly attributed their voice‐hearing to loneliness and social isolation. In turn, however, voices, irrespective of being positive or negative, could make social interactions more difficult as they can have a negative effect on concentration, leading to more social isolation and in some cases young people becoming more dependent upon their voices, as a result (Parry & Varese, 2020).

Collectively, there is only limited research that draws from cognitive models of voice‐hearing to investigate potential psychological determinants of distress in young people with this experience (Cavelti et al., 2019, 2020) or that adopts a relating perspective in understanding voice‐related distress in youth (Parry et al., 2020; Parry & Varese, 2020). This gap in the literature impacts on the development of appropriate psychological support and interventions for young people with distressing voice‐hearing. The current study aimed to examine some well‐established links in the cognitive model of voice‐hearing found in adults, while adopting a relating framework of understanding voice‐related distress, in a clinical sample of young people in Child and Adolescent Mental Health Services (CAMHS) and Early Intervention in Psychosis (EIP) services within the UK's National Health Service (NHS).

**FIGURE 1:**
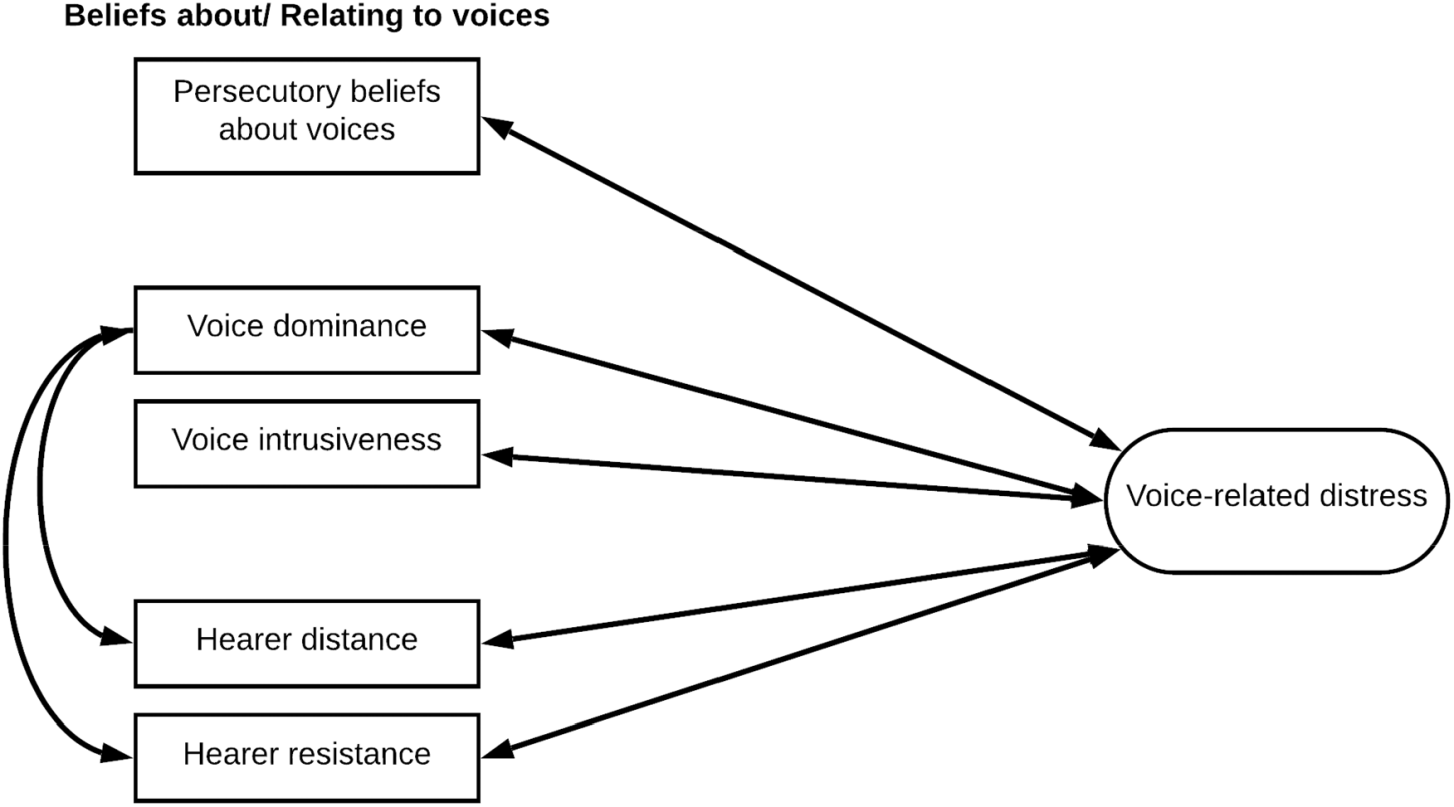
Hypothesised model of voice‐hearing in youth

It was hypothesised that voice dominance and intrusiveness, hearer distance and resistance to voices would be related to voice‐related distress (hypothesis 1) and that hearer distance from the voices and using a resistance mode of responding will be related to voice dominance (hypothesis 2). Furthermore, it was expected that persecutory beliefs about the voices would be related to voice‐related distress, independent from other voice characteristics such as their content, frequency and loudness (hypothesis 3). The main hypothesised relationships are represented diagrammatically in Figure 1.

Secondary aims of this study focused on comparing young people's relating to voices and relating to social others. Specifically, it was predicted that relating styles adopted with social others would be reflected in young people's relationship with voices, using measures of negative relating to social others and voices that have been previously administered in adult studies (Hayward, 2003; Hazell et al., 2018) (hypothesis 4). It was further hypothesised that hearer dependence to voices will be associated with lower social connectedness and belongingness (hypothesis 5).

## METHODS

### Design

This was a cross‐sectional, observational survey study using interviews and questionnaires with young people who hear voices. Ethics approval was provided by the London‐Brighton & Sussex Research Ethics Committee (reference number: 17/LO/2078).

### Participants

Considering that most bivariate correlations between variables of interest in the adult literature on the cognitive‐interpersonal model of voice‐hearing are of medium or large effect sizes (*r* ≥ .46) (Chadwick et al., 2000; Cole et al., 2017; Hayward, 2003; Hayward et al., 2008; Peters et al., 2012; Vaughan & Fowler, 2004), a sample of 34 voice‐hearers was deemed adequate to identify such effects (Hulley et al., 2013).

Participants were recruited from CAMHS and EIP services within a mental health NHS trust in South East England. Recruitment took place via referrals from mental health practitioners following young people's verbal consent or via self‐referrals. Inclusion criteria were as follows: (1) presence of voices for at least 3 months, (2) presence of voices within the past week, (3) aged 14–18 years at referral and (4) capacity to provide written, informed consent. Exclusion criteria were as follows: (1) voice‐hearing attributed to an organic illness or acute intoxication, solely to drug use or hypnagogic/ hypnopompic experiences, (2) insufficient English language ability for purposes of providing informed consent and completing assessment measures, (3) moderate or severe learning disability, (4) immediate risk to self or others or (5) voice‐hearing of little clinical significance, for example one's name being called and noises.

Written informed consent was provided by all participants. Where the participant was under 16 years of age, a person with parental responsibility also gave consent. All data were collected in multiple assessment meetings completed within one calendar month.

### Measures

#### Demographic measures

Participants completed self‐report questionnaires on demographics, diagnosis, current medication and details on psychological therapy for distressing voices or other mental health difficulties.

#### Clinical measures

The Comprehensive Assessment of At‐Risk Mental States—Short form (CAARMS; Yung et al., 2005), a semi‐structured interview was used to determine the presence of ultra‐high risk for psychosis or a first episode of psychosis status. The Structured Clinical Interview for Axis‐I DSM‐IV Disorders (SCID‐I‐RV; First et al., 2002), modules B (Psychotic symptoms) and C (Psychotic disorders) were rated for participants reaching the CAARMS psychosis threshold. The Mini International Neuropsychiatric Interview (MINI) for psychotic disorders studies (Version 7.0.2), a diagnostic interview based on the DSM‐V criteria, was completed to capture any research diagnosis for major psychiatric disorders. The Beck Depression Inventory‐II (BDI‐II, Beck et al., 1996), a 21‐item self‐report measure, captured the presence and severity of depressive symptoms with an overall score, with higher scores indicating higher levels of depressive symptoms. The Beck Anxiety Inventory (BAI, Beck & Steer, 1993), a 21‐item self‐report measure, captured the presence and severity of symptoms of anxiety, using the sum of its items, with higher scores representing higher levels of anxiety. Both BDI‐II and BAI have demonstrated good psychometric properties in adolescent studies (Krefetz et al., 2002, 2003; Osman et al., 2002; Steer et al., 1998), including this study (BAI, α = .93; BDI, α = .88).

#### Social relating measures

Three self‐report questionnaires were used to capture social relating. The shortened 48‐item Person's Relating to Others Questionnaire (PROQ‐3; Birtchnell et al., 2013) captured negative relating across eight subscales. These represented negative states of relatedness, described using two intersecting axes: that of proximity, referring the degree to which one needs to become involved with or separated from others, with polarities of ‘closeness’ and ‘distance’, and that of power, referring to the degree to which one chooses to exercise power over others or permit others to exercise their power over oneself, with polarities of ‘upperness’ and ‘lowerness’. This creates the following subscale names: upper neutral (UN), upper close (UC), neutral close (NC), lower close (LC), lower neutral (LN), lower distant (LD), neutral distant (ND) and upper distant (UD). Overall negative relating was also calculated as the sum of all subscale scores. The PROQ‐3 has acceptable internal consistency (α > .70 for all scales) and its eight‐factor structure is supported by factor analysis in young adults (Birtchnell et al., 2013). PROQ‐3 demonstrated acceptable internal consistency in the present sample (α > .68 for all scales). The 8‐item Social Connectedness Scale (mSCS; adapted from Lee & Robbins, 1995) captured sense of connectedness and a subscale from the Social Comparison Scale (SCS; Allan & Gilbert, 1995) measured perceived social belongingness in comparison with social others. In both measures, total scores were calculated with higher scores representing higher social connectedness and belongingness respectively. The mSCS has demonstrated good internal consistency (α = .91; Phillips et al., 2019) and convergent validity in young people (Lee et al., 2001). SCS has also demonstrated good validity and reliability in adolescents (α = .91) (Murphy et al., 2015). In the current study, the Cronbach's alpha scores for both scales indicated excellent internal consistency (mSCS, α = .93; SCS, α = .92).

#### Voice‐hearing measures

Three self‐report scales were used to measure voice‐hearing. The 11‐item Psychotic Symptom Rating Scales–Auditory Hallucinations Scale (PSYRATS‐AH; Haddock et al., 1999) captured symptom severity in the past week. Based on structural equation modelling analysis, the subscales measured distress (amount and degree of negative content, amount and intensity of distress, controllability), frequency (frequency, duration and disruption), attribution (location and beliefs about origin of voices) and loudness (Woodward et al., 2014), scored as the total scores of the subscale items. The subscales used in this study have demonstrated good reliability (intra‐class correlation coefficient > .93 for distress and > .87 for frequency) (Woodward et al., 2014), and they have been used in adult (e.g. Badcock et al., 2020; Craig et al., 2018; du Sert et al., 2018; Hayward et al., 2017, 2018; Jongeneel et al., 2018; Lincoln et al., 2021; Paulik et al., 2019, 2018) and youth research (Cavelti et al., 2019). In this study, internal consistency of PSYRATS‐AH subscales was acceptable (α > .66).

Negative content was also calculated as the mean score of the amount and degree of negative content items. Additional questions about familiarity of voice identity, synchronicity of voices, form of address (first, second and/or third person) and situation that trigger voices were taken from the Auditory Vocal Hallucination Scale (AVHRS; Jenner & van de Willige, 2002) and Manchester Voices Inventory for Children (MAVIC; Parry & Varese, 2020) to better capture young people's voice‐hearing experience.

The 35‐item Beliefs about Voices Questionnaire‐Revised (BAVQ‐R; Chadwick et al., 2000) captured persecutory (malevolence and omnipotence) beliefs about voices and resistive responses to voices, combining behavioural and emotional modes of response (Strauss et al., 2018), in the past week. Subscale scores were calculated as the mean score of their items. A study with young people has found adequate internal consistency estimates for BAVQ‐R (α > .71; Cavelti et al., 2019). In this study, both subscales used demonstrated good internal consistency (Persecutory beliefs, α = .89; Resistive responses, α = .83). The 28‐item Voice and You (VAY) (Hayward et al., 2008) was administered to record the interrelating between the participants and their predominant voice, with subscales capturing voice dominance, voice intrusiveness, hearer distance and hearer dependence to the voices. Scores for each subscale were calculated as the item total. The VAY has good internal consistency for all scales (α > .75; Hayward et al., 2008), which was supported in the present sample (α > .81).

### Data analysis

All analyses were carried out using SPSS (Version 25, IBM Corp., 2017). Bivariate correlations were conducted between relating (VAY voice dominance, voice intrusiveness, hearer distance and hearer dependence) and responding to voices (BAVQ resistance), persecutory beliefs about voices (BAVQ Persecutory beliefs) and voice‐related distress (PSYRATS distress). To investigate whether social relating was associated with relating to voices, bivariate correlations between relating to voices (VAY hearer distance and hearer dependence) and relating to social others (PROQ‐3 relating subscales, mSCS social connectedness and SCS social belongingness) were carried out. Some of the subscales of the PROQ‐3 (UN, NC, LC, ND subscales and total score), PSYRATS (distress, negative content) and the VAY (voice dominance and hearer dependence) showed kurtosis and/or skewness issues. Any analyses including these variables were conducted using Spearman's rho correlations.

To control for the effect of covariates, partial correlations were used. Within analyses exploring factors associated with voice‐related distress (PSYRATS distress), depression (BDI‐II total) and anxiety (BAI total), levels were considered as covariates to explore the unique contribution of these factors to voice‐related distress. Persecutory beliefs about voices (BAVQ‐R Persecutory Beliefs) were also controlled for to investigate the unique relationship between relating to voices (VAY subscales) and voice‐related distress. A partial correlation between persecutory beliefs about voices and voice‐related distress was conducted, controlling for voice characteristics (PSYRATS negative content, frequency and loudness). Age was tested as a potentially significant demographic covariate of all assessed associations.

The highest rate of missing cases was 11.8% (*N* = 4) for depression levels (BDI‐II total). Mann–Whitney U tests, independent samples t‐tests and Fisher's exact tests did not find any differences between the completers and non‐completers of any variables, *p*s > .001 (Bonferroni corrected critical *p*‐value). Analysis was carried out with the original data using available‐case analysis.

## RESULTS

### Sample characteristics

Table 1 presents the demographic and self‐reported clinical characteristics of the sample. In total, *n* = 28 young people were recruited from CAMHS and *n* = 6 from EIP services, with average age 16.28 years (*SD* = 1.09). The majority of participants identified as female (*n* = 25; 73.53%) and White British (*n* = 29; 85.29%) (see Table S1 and Table S2 for additional information).

**TABLE 1:** Sample characteristics and descriptive statistics (*N* = 34)

Sample characteristic	*N* (%)	*M* (min – max; *SD*)
Age		16.28 (14–18.95; 1.09)
Gender		
Male	7 (20.59)	
Female	25 (73.53)	
Other	2 (5.88)	
Identified as transgender	3 (8.82)	
Sexual orientation		
Heterosexual	15 (44.12)	
Lesbian	2 (5.88)	
Bisexual	10 (29.41)	
Other term	6 (17.65)	
Prefer not to say	1 (2.94)	
Ethnicity		
White British	29 (85.29)	
White Other	2 (5.88)	
Other	3 (8.82)	
Employment status^a^		
Student	31 (91.18)	
Employed part‐time (paid)	9 (26.47)	
Any self‐reported MH diagnosis	24 (70.59)	
Self‐reported diagnosis of Psychosis	5 (14.71)	
Taking any MH medication	24 (70.59)	
Taking antipsychotic medication	17 (50)	
Having received any psychological therapy	31 (91.18)	
Having received any psychological therapy for voices^b^	8 (25)	
Type of MH service		
CAMHS	28 (82.35)	
EIP	6 (17.65)	
Years since voice onset		4.79 (0.58–15; 3.88)
Number of voices^c^		36.06 (1–1000; 171.21)
Have a main voice^d^	25 (75.76)	

*Note*: Categories with count of *N* = 1 were suppressed to protect participant anonymity.

Abbreviations: CAMHS, Child and Adolescent Mental health services; EIP, Early Intervention in Psychosis services; MH, Mental health; *M*, mean; *N*, number of participants; *SD*, standard Deviation.

^a^ Multiple responses were allowed, with *N* = 7 reporting two employment statuses.

^b^ Missing *N* = 2.

^c^ Median = 2.

^d^ Missing *N* = 1.

Thirty‐one young people (91.18%) scored over psychotic threshold on the CAARMS. Using SCID‐I‐RV for Psychotic disorders, *n* = 20 (58.82%) met criteria for Psychotic Disorder Not Otherwise Specified, *n* = 8 (23.53%) for Schizophrenia, *n* = 2 for Schizoaffective disorder (5.88%) and *n* = 1 (2.94%) did not meet criteria for any SCID‐I‐RV diagnosis. The most commonly endorsed MINI diagnostic categories were past Major Depressive Episode (*n* = 28, 87.50%), past Panic Disorder (*n* = 19, 61.29%), current Panic Disorder (*n* = 12; 38.71%) and current Social Anxiety (*n* = 19, 61.29%) (see Table S3 for further details).

Descriptive statistics for all study variables are illustrated in Table 2.

**TABLE 2:** Descriptive statistics for main study variables in the study sample (*N* = 34)

Sample characteristic	*M* (min – max; *SD*)
BDI‐II total	38.13 (7–58; 11.58)
BAI total	33.59 (6–58; 14.31)
PROQ‐3 – UN	6.09 (1–15; 4.23)
PROQ‐3 – UC	7.27 (0–15; 4.83)
PROQ‐3 – NC	9.06 (2–15; 4.55)
PROQ‐3 – LC	11.97 (0–15; 3.96)
PROQ‐3 – LN	6.64 (0–15; 4.34)
PROQ‐3 – LD	7.52 (0–15; 3.78)
PROQ‐3 – ND	10.18 (0–15; 4.26)
PROQ‐3 – UD	7.09 (0–15; 6.43)
PROQ‐3 Overall	65.82 (28–117; 17)
SCS Belongingness	10.81 (3–22;5.40)
mSCS total	21.90 (8–46; 10.56)
PSYRATS Frequency	7.21 (2–10; 2.40)
PSYRATS Distress	15.26 (5–19; 3.67)
PSYRATS Loudness	2.79 (1–4; 1.09)
PSYRATS Negative Content	3.29 (0–4; 0.97)
BAVQ‐R Persecutory Beliefs	1.96 (0.25–3; 0.66)
BAVQ‐R Resistance	1.74 (0.11–2.89; 0.68)
VAY Voice dominance	15.74 (2–21; 5.40)
VAY Voice intrusiveness	8.65 (0–15; 4.78)
VAY Hearer distance	13.24 (1–21; 5.97)
VAY Hearer dependence	7.34 (0–26; 6.72)

Abbreviations: BAI, Beck Anxiety Inventory; BAVQ‐R, Beliefs about Voices Questionnaire‐Revised; BDI‐II, Beck's Depression Inventory‐II; *M*, mean; mSCS, Social Connectedness Scale; *N*, number of participants; PROQ‐3, shortened Person's Relating to Others Questionnaire; PSYRATS, Psychotic Symptom Rating Scales; SCS, Social Comparison Scale; *SD*, standard deviation; VAY, The Voice and You.

#### Hypothesis testing

Hypothesis 1 Voice dominance and intrusiveness, hearer's distance and resistance mode of responding will be related to voice‐related distress.

Voice dominance, voice intrusiveness and a resistance mode of responding were significantly correlated with voice‐related distress (*r*_s_ = .47, *p* = .005, *r*_s_ = .42, *p* = .014, *r*_s_ = .40, *p* = .021 respectively). However, hearer distance was not related to voice‐related distress (*r*_s_ = .25, *p* = .157). After controlling for BDI‐II depression and BAI anxiety levels individually to isolate the effect of relating variables on voice‐related distress, only voice dominance was significantly related to voice‐related distress with *r*_*s*_ = .42, *p* = .024 and *r*_*s*_ = .39, *p* = .024, respectively.

To test for the unique contribution of relating to voices (VAY subscales) to voice‐related distress, persecutory beliefs about voices (BAVQ‐R Persecutory beliefs) were controlled for with or without levels of BDI‐II depression and BAI anxiety levels. Results showed that none of the hypothesis 1 relationships remained significant (*p*s > .05) when controlling for the level of persecutory beliefs, regardless of whether BAI and BDI‐II levels were also included as covariates.

After controlling for the effect of age, voice dominance, voice intrusiveness and hearer resistance to voices remained significantly associated with voice‐related distress.

Hypothesis 2 Hearer's distancing from the voices and using a resistance mode of responding will be related to voice dominance.

Hearer distance (*r*_s_ = .69, *p* < .001) and resistance (*r*_s_ = .54 *p* = .001) were significantly correlated with voice dominance.

Controlling for age did not have any significant impact on the results.

**FIGURE 2:**
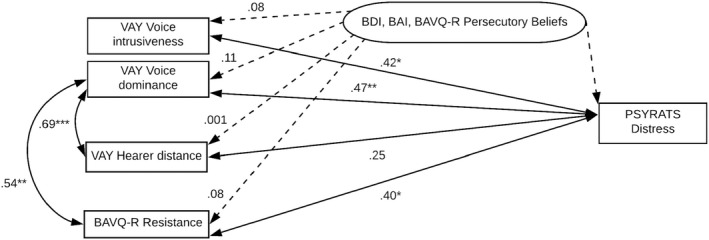
Representation of the relationships between voice dominance and intrusiveness, hearer distance, resistance and voice‐related distress, *N* = 34. Dotted lines represent partial Spearman's rank correlations, controlling for anxiety, depression and persecutory beliefs (*p < .05; **p < .01; ***p < .001). Partial correlations included *N* = 23 due to pairwise deletion. Any paired associations between BAVQ‐R resistance and other variables included *N* = 33

Figure 2 provides an overview of the tested relationships in hypotheses 1 and 2.

Hypothesis 3 Persecutory beliefs about the voices will be related to voice‐related distress, independent from other voice characteristics such as their content, frequency and loudness.

**FIGURE 3:**
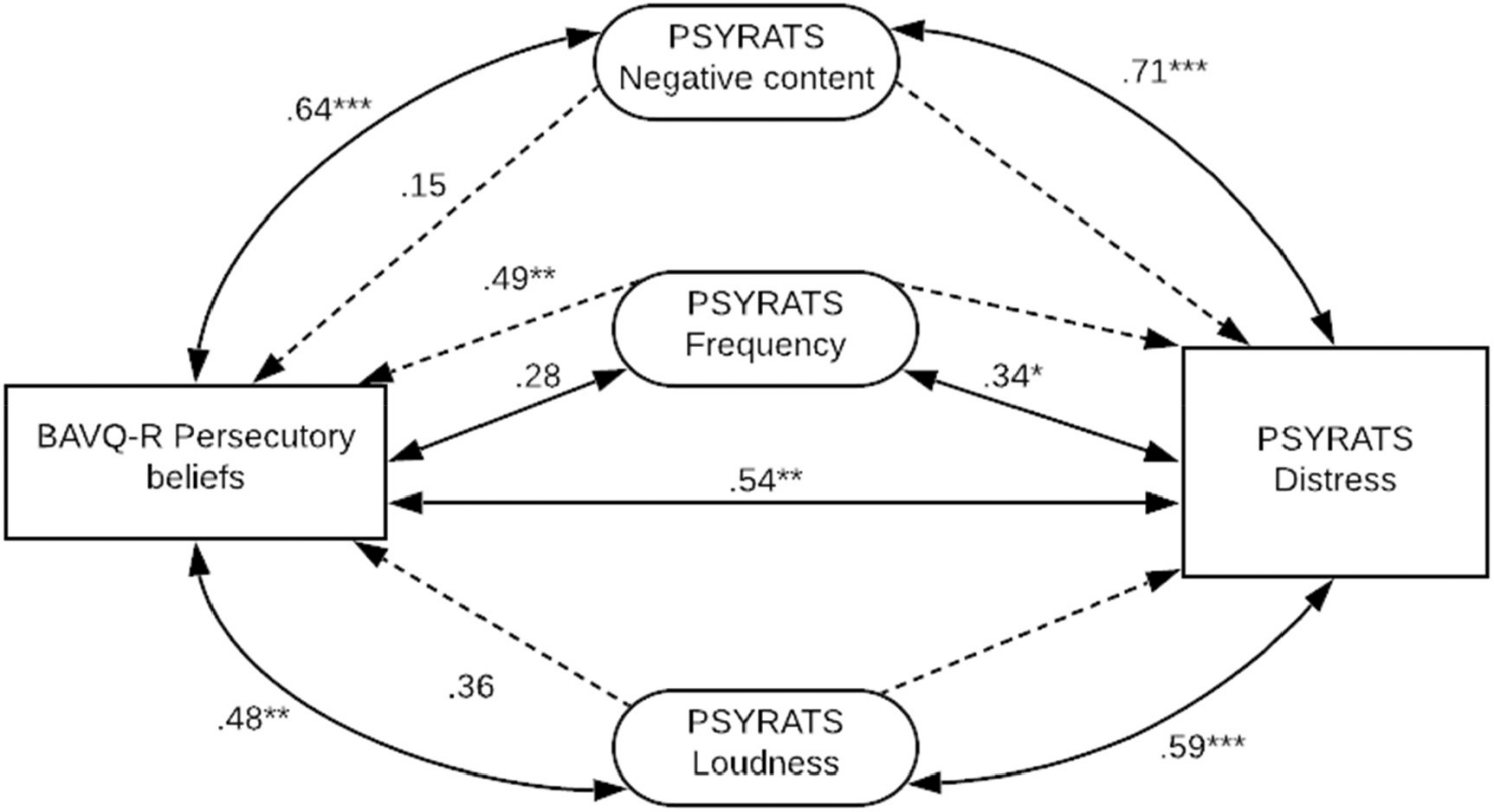
Representation of the relationships between persecutory beliefs, voice characteristics and voice‐related distress. Dotted lines represent partial Spearman's rank correlations, controlling for negative content, frequency and loudness individually (*p < .05; **p < .01; ***p < .001). Partial correlations included *N* = 28 due to pairwise deletion

Persecutory beliefs were significantly associated with voice‐related distress, *r*_*s*_ = .54, *p* = .002, *N* = 31. When controlling for voice frequency, persecutory beliefs were still significantly related to voice‐related distress, *r*_*s*_ = .49, *p* = .006. In contrast, when loudness or negative voice content were factored in, the association between persecutory beliefs and voice‐related distress was no longer significant (*r*_*s*_ = .36, *p* = .052 and *r*_*s*_ = .15, *p* = .418 respectively). Adjusting for all three voice characteristics at the same time resulted in a non‐significant association between persecutory beliefs and voice‐related distress (*r*_*s*_ = −.04, *p* = .827, *N* = 26) (Figure 3).

Adjusting for the effect of age did not impact on the significance of the reported relationships.

Hypothesis 4 Relating from a position of distance to voices will be associated with distant relating styles with others, while relating from a position of dependence to voices will be associated with relating dependently with social others.

Contrary to the hypothesis, correlations between the PROQ‐3 subscales and VAY hearer distance and dependence showed that neutral close relating (fear of separation and of being alone) was related to hearer distance (*r*_*s*_ = .37, *p* = .032). Additionally, neutral distant (suspicious, uncommunicative and self‐reliant) and upper distant types of relating (sadistic, intimidating and tyrannising) to others were significantly associated with hearer dependence, *r*_*s*_ = .494, *p* = .005 and *r*_*s*_ = .56, *p* = .001, respectively.

When controlling for the effect of age, the associations remained significant.

Hypothesis 5 Relating from a position of dependence with the voices will be negatively associated with social connectedness and social belongingness.

Non‐parametric associations were conducted between social connectedness (mSCS Total) and social belongingness (SCS belongingness) with VAY hearer distance and dependence. Results suggested that relating dependently with voices was associated with lower perceived social belongingness and connectedness, *r*_*s*_ = −.45, *p* = .014 and *r*_*s*_ = −.53, *p* = .004, respectively.

Controlling for the effect of age did not have a significant impact on the reported associations.

A correlation matrix for all study variables, a summary of hypotheses testing outcomes and the updated cognitive‐interpersonal model of voice‐hearing in youth can be found in Supplementary material (Tables S4 and S5 and FigureS1).

## DISCUSSION

This study explored the extent to which the cognitive‐interpersonal model of voice‐hearing, developed with adults, could facilitate an understanding of the voice‐hearing experienced by young people. The model was found to have some relevance with regard to the influence of appraisals and negative content of voices on voice‐related distress. Inconsistencies were evident with regard to the influence of voice loudness and the lack of associations between relating to voices and social relating.

### Relating to voices and voice‐related distress

The finding that the more dominant and intrusive the voices were perceived to be, the greater the distress that was experienced is in line with previous studies with adults (Hayward et al., 2008; León‐Palacios et al., 2015; Sorrell et al., 2010; Vaughan & Fowler, 2004). Moreover, that controlling for persecutory beliefs about voices renders the associations between voice dominance and voice intrusiveness to distress almost non‐existent is consistent with evidence that persecutory beliefs about voices potentially moderate or mediate the association between relating styles and voice‐related distress (Sorrell et al., 2010) and general distress (León‐Palacios et al., 2015). However, contrary to expectations, this was not the case for the association between hearer distance and voice‐related distress. It could be that resistance represents a mode of responding to voices rather than a relating position, and thus, the hearer distance items might not feel relevant to young people's experience. This seems to be consistent with young people's narratives of negative, controlling, ‘haunting’ voices lacking a relational reciprocity compared with accounts of pleasant voices (Parry & Varese, 2020).

The correlation of greater resistance with higher levels of voice‐related distress was in accordance with Chadwick and Birchwood (1994) who suggested that persecutory, dominant voices are resisted, which leads to further voice occurrence and distress. Indeed, early studies showed problem‐focused coping strategies, such as resistance (which includes strategies to inhibit voices), could be ineffective in reducing distress (Farhall & Gehrke, 1997) or could even increase distress (Singh et al., 2003). Peters et al. (2012) supported these findings by indicating an association between hearer resistance and voice‐related distress, while others have found an association of resistance with general distress (depression and anxiety) (Chadwick et al., 2000; Fannon et al., 2009; Morris et al., 2014).

### Beliefs about voices, voice characteristics and negative content: Links with voice‐related distress

The present finding that persecutory beliefs about voices were a significant determinant of distress even after controlling for voice characteristics is consistent with findings from the adult literature (Birchwood & Chadwick, 1997; Peters et al., 2012; Van der Gaag et al., 2003). However, the finding that the persecutory beliefs‐distress association became non‐significant when adjusting for loudness is distinct to the current young sample, since appraisals of voices seem to consistently contribute to distress over and above their physical characteristics amongst adults (Birchwood & Chadwick, 1997; Peters et al., 2012; Van der Gaag et al., 2003). It might be that louder voices are more distressing or lead to greater life disruption for young people (Parry & Varese, 2020), which in turn could be strengthening beliefs about the persecutory nature of voices. It is notable that smaller numbers of adults report voices shouting (4%; McCarthy‐Jones et al., 2014) compared with the substantial minority of young people in the present study (38%) reported hearing shouting voices. Thus, voices in young people might differ in their phenomenology from those in adults, at least in the present sample.

Controlling for the effect of negative voice content rendered the association between persecutory beliefs and voice‐related distress almost non‐existent. Chadwick and Birchwood (1994) have previously emphasised the importance of voice content, stating that content can provide evidence in support of particular beliefs. Negative content might also predispose hearers towards beliefs about the persecutory nature of voices (Van der Gaag et al., 2003). Cole et al. (2017) stressed the need to include voice content as a determinant of distress in future studies, as it may be linked with distress, not only via the mediating role of voice beliefs, but also directly. Recent youth research (Cavelti et al., 2019) has shown negative content to correlate moderately to strongly with general distress. In the present study, negative content was more strongly related to voice‐related distress than beliefs about voices, supporting its significant contribution to distress.

### Relating to voices and relating to others: More different than similar?

Contrary to predictions, relating to voices from a position of distance was correlated with adopting a close (‘clinging’) style of relating to other people, whereas relating to voices from a position of dependence was associated with being distant towards social others. Therefore, for young people, unlike adults (Birchwood et al., 2004; Hayward, 2003), relating to voices appears to be the inverse of relating styles enacted with social others. It could be that young people are less likely than adults to experience a sense of having a relationship with their voices, perhaps as a way of preserving their self‐hood and/or a way of rejecting the stigma attached to voice‐hearing (Garrett & Silva, 2003). However, it is worth noting that, although negative relating with voices and social others did not follow the same direction, there was still a link between being dependent on the voices and distant relating to social others. This agrees with findings from adult patients with schizophrenia spectrum diagnoses where engagement and immersion in the voice‐hearer relationship was linked with poor communication and withdrawal from social others (Favrod et al., 2004). Thus, it could be speculated that seeking approval from and closeness with the voices is related to distancing oneself from social others and feeling less connected with the social world, with voices functioning as a replacement for depleted social networks (Mawson et al., 2011) or difficult social relationships (Parry & Varese, 2020).

### Clinical implications

The findings from this study offer meaningful clinical implications. The identification of voice‐hearing in youth warrants clinicians' attention, and it should be followed by a detailed assessment of their experience, exploring potential factors that might be linked with voice‐related distress, such as beliefs about voice intent and power, any evidence (e.g. negative content and loudness) for this interpretation and responses to voices. Furthermore, most young people who seek help for hearing voices have reported feeling distressed by the experience (Maijer et al., 2014, 2017), indicating a need for support. Although a few studies have explored the feasibility of Cognitive Behavioural Therapy—informed interventions for young people with distressing voices (Hayward et al., 2022; Maijer et al., 2020), or the acceptability and potential clinical utility of such protocols for distressing psychotic experiences in youth more broadly (Jolley et al., 2018), these interventions were not based on evidence specific to distress factors of voice‐hearing in youth. Hence, this study provides preliminary evidence suggesting that psychological interventions targeting beliefs about voices, relating to voices and maladaptive responses to voices might be beneficial in young people. Voice loudness and negative content were important distress contributors and might be markers for more severe presentations and stronger beliefs about the persecutory nature of the voices. Exploring negative voice content in therapy could be beneficial for young people, especially in identifying connections of content to past relationships or experiences, as this could strengthen the adoption of less pathologising voice‐hearing narratives (Parry & Varese, 2020). Lastly, when designing a support plan for young voice‐hearers, particular attention should be paid to those who might seem dependent on their relationship with voices as this could contribute to issues in their social life, such as social withdrawal and feelings of disconnect (Mawson et al., 2011; Parry & Varese, 2020).

### Limitations and future directions

This study had several limitations. First, the sample size was small, so it cannot be concluded that the non‐significant findings reflect a true absence of associations. Second, there was no correction for multiple testing which increased the risk of false‐positive results. Hypotheses were theory‐driven, yet replication with a larger sample is encouraged. Third, due to the cross‐sectional design of the study, causal inferences regarding the relationship between the variables of interest cannot be drawn and interpretation of results was based on previous adult findings. Moreover, the operationalisation of voice‐related distress is based on a factor analysis by Woodward et al. (2014), which combines items concerning the amount and degree of voice‐related distress with items concerning negative content and control over the experience. Thus, the significant relationship between negative content (amount and degree) and distress might be influenced by the overlap between these two variables. Additionally, voice‐related distress has not been operationalised consistently in the literature. For example, Cole et al. (2017) used one item concerning the intensity of voice‐related distress from the Hamilton Program for Schizophrenia Voices Questionnaire (Van Lieshout & Goldberg, 2007), similar to studies with young people that used single‐item scores about distress intensity, that were of self‐report questionnaires assessing psychotic‐like or unusual experiences (Brink et al., 2020; Jolley et al., 2017). Thus, the present findings might not be directly comparable to other studies due to methodological differences in the operationalisation of distress. Furthermore, some of the measures used have not been validated in adolescent samples (PROQ‐3, VAY, PSYRATS‐AH and BAVQ‐R). Although in this study, they demonstrated acceptable internal consistency, they need to be psychometrically validated in youth to further support their relevance and use in this group.

Future investigations of the links of the cognitive‐interpersonal model, using a longitudinal design and a larger sample size, are needed to allow the exploration of the dynamic relationship between psychological factors and voice‐related distress in youth. Considering that young people with distressing voices might be found outside mental health services, an investigation of cognitive‐interpersonal model of voice‐hearing could be expanded to community samples, to further examine potential factors that contribute to a need for care.

## CONCLUSIONS

This study provided preliminary evidence that persecutory beliefs about voices, resistive responses to voices and voice loudness and negative content might be significant contributors of distress in young people who experience voice‐hearing and co‐occurring mental health difficulties. Clinicians working with these young people should assess and potentially target these factors within psychological interventions, paying additional attention to the relating between the young person and their voices as it might be an index for social relating and connectedness difficulties.

## AUTHOR CONTRIBUTIONS

**Aikaterini Rammou:** Conceptualization; Data curation; Formal analysis; Funding acquisition; Investigation; Methodology; Project administration; Resources; Software; Validation; Visualization; Writing – original draft; Writing – review and editing. **Clio Berry:** Conceptualization; investigation; methodology; resources; supervision; validation; visualization; writing – review and editing. **David Fowler:** Conceptualization; methodology; supervision; writing – review and editing. **Mark Hayward:** Conceptualization; funding acquisition; methodology; supervision; writing – original draft; writing – review and editing.

## CONFLICT OF INTEREST

All authors declare no conflict of interest.

## Supporting information


Figure S1

Table S1

Table S2

Table S3

Table S4

Table S5

## Data Availability

The data that support the findings of this study are available on request from the corresponding author. The data are not publicly available due to privacy or ethical restrictions.
