# Supplementary material for: Distress factors of voice‐hearing in young people and social relating: Exploring a cognitive‐interpersonal voice‐hearing model
Source: Psychol Psychother. 2022 Jun 30;95(4):939–57. doi: 10.1111/papt.12411 (PMC9795969; doi:10.1111/papt.12411)

**Supplementary Material Figure 1.** *The updated model of voice-hearing in youth. Black solid lines represent statistically significant bidirectional associations between constructs. Grey solid lines represent bidirectional associations between constructs that were not supported, contrary to the study hypotheses. Dotted lines represent partial correlations. Dotted grey lines indicate that the conceptual predictor-outcome association did not remain significant after controlling for the covariates. Dotted black lines indicate that the association between the conceptual predictor and outcome remained significant after controlling for the covariates*.


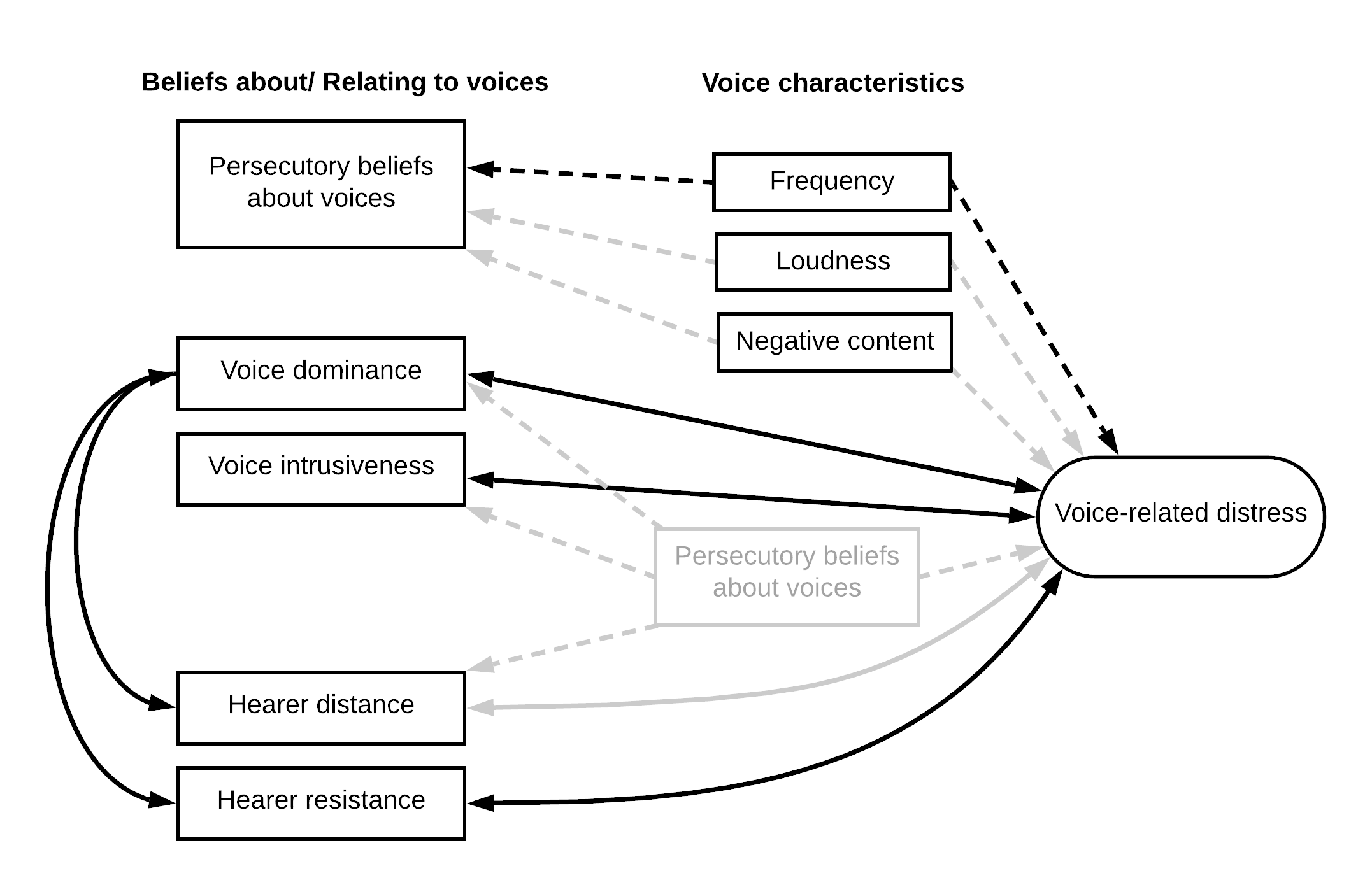

Supplement: Supplementary file 1 — Figure S1 [file PAPT-95-939-s002.docx]
