# Supplementary material for: Distress factors of voice‐hearing in young people and social relating: Exploring a cognitive‐interpersonal voice‐hearing model
Source: Psychol Psychother. 2022 Jun 30;95(4):939–57. doi: 10.1111/papt.12411 (PMC9795969; doi:10.1111/papt.12411)
Supplement: Supplementary file 2 — Table S1 [file PAPT-95-939-s005.docx]

Supplementary Material Table 1. *Additional sample characteristics and descriptive statistics (N = 34).*

| Sample characteristic | *N* (%) | *M* (Min- Max; *SD*) |
| --- | --- | --- |
| Marital status |  |  |
| Single | 23 (67.65) |  |
| In a long-term relationship/Cohabitating | 10 (29.41) |  |
| Prefer not to say | 1 (2.94) |  |
| Country of birth |  |  |
| UK or Northern Ireland | 31 (91.18) |  |
| Other | 3 (8.82) |  |
| Educational level |  |  |
| None | 22 (64.71) |  |
| GCSEs or equivalent | 9 (26.47) |  |
| A level or equivalent | 3 (8.82) |  |
| Limited day-to-day activities due to disability |  |  |
| Yes | 6 (17.65) |  |
| No | 28 (82.35) |  |
| Religion |  |  |
| Agnostic | 8 (23.53) |  |
| Atheist | 17 (50.0) |  |
| Christian | 7 (20.59) |  |
| Other | 1 (2.94) |  |
| Prefer not to say | 1 (2.94) |  |
| *Note.* *M* = Mean; *SD* = Standard Deviation. Categories with count of *N* = 1 were suppressed to protect participant anonymity. | | |
