# Supplementary material for: Distress factors of voice‐hearing in young people and social relating: Exploring a cognitive‐interpersonal voice‐hearing model
Source: Psychol Psychother. 2022 Jun 30;95(4):939–57. doi: 10.1111/papt.12411 (PMC9795969; doi:10.1111/papt.12411)
Supplement: Supplementary file 3 — Table S2 [file PAPT-95-939-s001.docx]

Supplementary Material Table 2. *Additional descriptive statistics of voice-hearing characteristics in the sample (N = 34).*

| Sample characteristic | *N* (valid %) |
| --- | --- |
| Voice synchronicity^b^ |  |
| Always one voice | 10 (30.30) |
| Speaking separately | 4 (12.12) |
| Speaking simultaneously | 19 (57.58) |
| Voice duration |  |
| Few seconds | 4 (11.76) |
| Several minutes | 9 (26.47) |
| At least an hour | 3 (8.82) |
| Hours at a time | 18 (52.94) |
| Voice frequency |  |
| At least once a week | 7 (20.59) |
| At least once a da | 8 (23.53) |
| At least once an hour | 4 (11.76) |
| Continuously or almost continuously | 15 (44.12) |
| Voice location |  |
| Inside head only | 8 (23.53) |
| Outside head, close to ears (+inside the head could be present) | 17 (50) |
| Outside head way from ears (+inside the head or close to ears could be present) | 7 (20.59) |
| Outside only | 2 (5.88) |
| Loudness |  |
| Lower than own voice | 4 (11.76) |
| Same as own voice | 12 (35.29) |
| Louder than own voice | 5 (14.71) |
| Shouting | 13 (38.24) |
| Beliefs about voice origin |  |
| Internally generated only | 14 (41.18) |
| <50% from external causes | 16 (47.06) |
| >50% (less than 100%) from external causes | 2 (5.88) |
| Externally generated only | 2 (5.88) |
| Amount of negative content |  |
| None | 2 (5.88) |
| Minority (>10 %<50%) | 5 (14.7) |
| Majority (>50%) | 13 (38.2) |
| All | 14 (41.2) |
| Degree of negative content |  |
| No negative content | 2 (5.88) |
| Personal verbal abuse,  comments on behaviour | 1 (2.94) |
| Personal verbal abuse,  relating to self-concept | 7 (20.59) |
| Personal threats to self/others or extreme instructions to harm self/others | 24 (70.59) |
| Amount of distress |  |
| Not at all | 1 (2.94) |
| <10% distressing | 2 (5.88) |
| <50% distressing | 8 (23.53) |
| >50% distressing | 12 (35.29) |
| Always distressing | 11 (32.35) |
| Intensity of distress |  |
| Not at all | 2 (5.88) |
| Slightly | 6 (17.65) |
| Moderate degree | 6 (17.65) |
| Very distressing | 18 (52.94) |
| Extremely distressing | 2 (5.88) |
| Disruption to life due to voices |  |
| No disruption | 3 (8.82) |
| Minimal | 15 (44.12) |
| Moderate | 16 (47.12) |
| Controllability of voices |  |
| Over majority of occasions | 3 (8.82) |
| Over half of occasions | 2 (5.88) |
| Over minority of occasions | 6 (17.65) |
| No control | 23 (67.65) |
| Timing |  |
| Hypnopompic/ Hypnagogic only | 0 |
| At all times | 34 (100) |
| Form of address^c^ |  |
| 1^st^ person | 10 (29.41) |
| 2^nd^ person | 34 (100) |
| 3^rd^ person | 14 (41.18) |
| Usual time of the day voices start |  |
| As soon as waking up | 2 (5.88) |
| Afternoon | 2 (5.88) |
| Evening | 4 (11.76) |
| Just before bed | 2 (5.88) |
| Any time | 24 (70.59) |
| Usual situation voices start |  |
| When alone | 5 (14.71) |
| Around a lot of people | 2 (5.88) |
| Always the same | 27 (79.41) |
| Familiarity of voice identity^c^ |  |
| Familiar | 9 (26.47) |
| Strange | 19 (55.88) |
| Sometimes/not sure | 9 (26.47) |
| Familiar, but not how they sound in person | 2 (5.88) |
| *Note.* ^a^ Median = 2; ^b^ missing *N* = 1; ^c^ Multiple responses allowed, “+” = “and”; *N* = Number of participants; *M* = Mean; *SD* = Standard Deviation. | |
