# Supplementary material for: Distress factors of voice‐hearing in young people and social relating: Exploring a cognitive‐interpersonal voice‐hearing model
Source: Psychol Psychother. 2022 Jun 30;95(4):939–57. doi: 10.1111/papt.12411 (PMC9795969; doi:10.1111/papt.12411)
Supplement: Supplementary file 4 — Table S3 [file PAPT-95-939-s003.docx]

Supplementary Material Table 3. *Descriptive statistics of clinical measures in the sample (N = 34).*

| Sample characteristic | *N* (Valid %) | *M* (Min- Max; *SD*) |
| --- | --- | --- |
| **UHR status** |  |  |
| Not at risk | 1 (2.94) |  |
| Attenuated Psychotic Symptoms | 2 (5.88) |  |
| Over psychotic threshold | 31 (91.18) |  |
| **SCID Psychotic disorders** |  |  |
| Not meeting criteria/Not applicable | 4 (11.76) |  |
| Schizophrenia | 8 (23.53) |  |
| Schizoaffective | 2 (5.88) |  |
| Psychotic Disorder Not Otherwise Specified | 20 (58.82) |  |
| **MINI diagnostic categories^a^** |  |  |
| MDE | 9 (28.13) |  |
| Past MDE | 28 (87.50) |  |
| Manic Episode | 0 |  |
| Past Manic Episode | 9 (28.13) |  |
| Hypomanic Episode | 0 |  |
| Past Hypomanic Episode | 1 (3.13) |  |
| Hypomanic Symptoms | 1 (3.13) |  |
| Past Hypomanic Symptoms | 0 |  |
| Panic Disorder | 12 (38.71) |  |
| Past Panic disorder | 19 (61.29) |  |
| Agoraphobia | 9 (29.03) |  |
| Social Anxiety | 19 (61.29) |  |
| Obsessive compulsive disorder | 6 (19.35) |  |
| Post-traumatic stress disorder | 7 (22.58) |  |
| Alcohol Use disorder 12 months | 7 (22.58) |  |
| Substance Use Disorder 12months | 7 (22.58) |  |
| Anorexia Nevrosa (Restricting) | 1(3.23) |  |
| Bulimia Nevrosa | 4 (12.90) |  |
| Binge Eating | 0 |  |
| Generalised Anxiety disorder | 2 (6.45) |  |
| *Note.* ^a^Missing to *N* = 2 from MINI MDE to Past Hypomanic Symptoms and *N* = 3 missing for the rest of MINI categories; *N* = Number of participants; *M* = Mean; *SD* = Standard Deviation; UHR = ultra high-risk for psychosis based on the Comprehensive Assessment of At-Risk Mental States; SCID = Structured Clinical Interview for Axis-I DSM-IV Disorders; MINI = Mini International Neuropsychiatric Interview; MDE = Major Depressive Episode. | | |
