# Supplementary material for: Distress factors of voice‐hearing in young people and social relating: Exploring a cognitive‐interpersonal voice‐hearing model
Source: Psychol Psychother. 2022 Jun 30;95(4):939–57. doi: 10.1111/papt.12411 (PMC9795969; doi:10.1111/papt.12411)
Supplement: Supplementary file 5 — Table S4 [file PAPT-95-939-s006.docx]

Supplementary Material Table 4. *Correlation Matrix for all study variables presenting correlation coefficients (N=34).*

| Variables | 1 | 2 | 3 | 4 | 5 | 6 | 7 | 8 | 9 | 10 | 11 | 12 | 13 | 14 | 15 | 16 | 17 | 18 | 19 | 20 | 21 | 22 | 23 |
| --- | --- | --- | --- | --- | --- | --- | --- | --- | --- | --- | --- | --- | --- | --- | --- | --- | --- | --- | --- | --- | --- | --- | --- |
| 1 Age | ___ |  |  |  |  |  |  |  |  |  |  |  |  |  |  |  |  |  |  |  |  |  |  |
|  |  |  |  |  |  |  |  |  |  |  |  |  |  |  |  |  |  |  |  |  |  |  |  |
| 2 BDI-II total | .04^a^ | ___ |  |  |  |  |  |  |  |  |  |  |  |  |  |  |  |  |  |  |  |  |  |
|  | *N* = 30 |  |  |  |  |  |  |  |  |  |  |  |  |  |  |  |  |  |  |  |  |  |  |
| 3 BAI total | -.28 ^a^ | .51^a **^ | ___ |  |  |  |  |  |  |  |  |  |  |  |  |  |  |  |  |  |  |  |  |
|  | *N* = 34 | *N* = 30 |  |  |  |  |  |  |  |  |  |  |  |  |  |  |  |  |  |  |  |  |  |
| 4 PROQ-3 – UN | .05 | .18 | -.28 | ___ |  |  |  |  |  |  |  |  |  |  |  |  |  |  |  |  |  |  |  |
|  | *N* = 33 | *N* = 29 | *N* = 33 |  |  |  |  |  |  |  |  |  |  |  |  |  |  |  |  |  |  |  |  |
| 5 PROQ-3 – UC | -.07^a^ | .22^a^ | .09^a^ | .32 | ___ |  |  |  |  |  |  |  |  |  |  |  |  |  |  |  |  |  |  |
|  | *N* = 33 | *N* = 29 | *N* = 33 | *N* = 33 |  |  |  |  |  |  |  |  |  |  |  |  |  |  |  |  |  |  |  |
| 6 PROQ-3 – NC | -.18 | .30 | .16 | .27 | .61^***^ | ___ |  |  |  |  |  |  |  |  |  |  |  |  |  |  |  |  |  |
|  | *N* = 33 | *N* = 29 | *N* = 33 | *N* = 33 | *N* = 33 |  |  |  |  |  |  |  |  |  |  |  |  |  |  |  |  |  |  |
| 7 PROQ-3 – LC | .08 | .34 | .41^*^ | -.02 | .23 | .21 | ____ |  |  |  |  |  |  |  |  |  |  |  |  |  |  |  |  |
|  | *N* = 33 | *N* = 29 | *N* = 33 | *N* = 33 | *N* = 33 | *N* = 33 |  |  |  |  |  |  |  |  |  |  |  |  |  |  |  |  |  |
| 8 PROQ-3 – LN | .14^a^ | .14^a^ | .48^a **^ | -.42^*^ | .10^a^ | -.09 | .39^*^ | ___ |  |  |  |  |  |  |  |  |  |  |  |  |  |  |  |
|  | *N* = 33 | *N* = 29 | *N* = 33 | *N* = 33 | *N* = 33 | *N* = 33 | *N* = 33 |  |  |  |  |  |  |  |  |  |  |  |  |  |  |  |  |
| 9 PROQ-3 – LD | .001^a^ | .11^a^ | .27^a^ | -.13 | .33^a^ | .10 | .32 | .65^a***^ | ___ |  |  |  |  |  |  |  |  |  |  |  |  |  |  |
|  | *N* = 33 | *N* = 29 | *N* = 33 | *N* = 33 | *N* = 33 | *N* = 33 | *N* = 33 | *N* = 33 |  |  |  |  |  |  |  |  |  |  |  |  |  |  |  |
| 10 PROQ-3 – ND | .05 | .50 ^**^ | .41^*^ | -.02 | -.07 | -.19 | .32 | .30 | .06 | ___ |  |  |  |  |  |  |  |  |  |  |  |  |  |
|  | *N* = 33 | *N* = 29 | *N* = 33 | *N* = 33 | *N* = 33 | *N* = 33 | *N* = 33 | *N* = 33 | *N* = 33 |  |  |  |  |  |  |  |  |  |  |  |  |  |  |
| 11 PROQ-3 – UD | -.13^a^ | .34^a^ | -.001^a^ | .50^**^ | .04^a^ | .12 | -.40 | -.29^a^ | -.50^a**^ | .52^**^ | ___ |  |  |  |  |  |  |  |  |  |  |  |  |
|  | *N* = 33 | *N* = 29 | *N* = 33 | *N* = 33 | *N* = 33 | *N* = 33 | *N* = 33 | *N* = 33 | *N* = 33 | *N* = 33 |  |  |  |  |  |  |  |  |  |  |  |  |  |
| 12 PROQ-3 overall | .11 | .48 ^**^ | .48^**^ | .31 | .66^***^ | .52^**^ | .51^**^ | .35^*^ | .32 | .46^**^ | .42^*^ | ___ |  |  |  |  |  |  |  |  |  |  |  |
|  | *N* = 33 | *N* = 29 | *N* = 33 | *N* = 33 | *N* = 33 | *N* = 33 | *N* = 33 | *N* = 33 | *N* = 33 | *N* = 33 | *N* = 33 |  |  |  |  |  |  |  |  |  |  |  |  |
| 13 SCS belongingness | .28^a^ | -.33^a^ | -.18^a^ | .13 | .02^a^ | .12 | -.48^**^ | -.17^a^ | -.11^a^ | -.48^**^ | -.20^a^ | -.23 | ___ |  |  |  |  |  |  |  |  |  |  |
|  | *N* = 32 | *N* = 28 | *N* = 32 | *N* = 32 | *N* = 32 | *N* = 32 | *N* = 32 | *N* = 32 | *N* = 32 | *N* = 32 | *N* = 32 | *N* = 32 |  |  |  |  |  |  |  |  |  |  |  |
| 14 mSCS total | -.16^a^ | -.64^a ***^ | -.29^a^ | -.02 | -.08^a^ | .09 | -.49^**^ | -.31^a^ | -.21^a^ | -.72^**^ | -.34^a^ | -.48^**^ | .49^a **^ | ___ |  |  |  |  |  |  |  |  |  |
|  | *N* = 30 | *N* = 26 | *N* = 30 | *N* = 30 | *N* = 30 | *N* = 30 | *N* = 30 | *N* = 30 | *N* = 30 | *N* = 30 | *N* = 30 | *N* = 30 | *N* = 29 |  |  |  |  |  |  |  |  |  |  |
| Variables | 1 | 2 | 3 | 4 | 5 | 6 | 7 | 8 | 9 | 10 | 11 | 12 | 13 | 14 | 15 | 16 | 17 | 18 | 19 | 20 | 21 | 22 | 23 |
| 15 PSYRATS Frequency | -.26^a^ | .11^a^ | .04^a^ | -.05 | -.01^a^ | .09 | -.05 | .20^a^ | .01^a^ | .02 | .10^a^ | .11 | -.32 ^a^ | .07^a^ | ___ |  |  |  |  |  |  |  |  |
|  | *N* = 34 | *N* = 30 | *N* = 34 | *N* = 33 | *N* = 33 | *N* = 33 | *N* = 33 | *N* = 33 | *N* = 33 | *N* = 33 | *N* = 33 | *N* = 33 | *N* = 32 | *N* = 30 |  |  |  |  |  |  |  |  |  |
| 16 PSYRATS Distress | -.27 | .25 | .34 | -.22 | -.15 | .06 | -.05 | .19 | .29 | .08 | -.07 | .04 | -.15 | -.04 | .34^*^ | ___ |  |  |  |  |  |  |  |
|  | *N* = 34 | *N* = 30 | *N* = 34 | *N* = 33 | *N* = 33 | *N* = 33 | *N* = 33 | *N* = 33 | *N* = 33 | *N* = 33 | *N* = 33 | *N* = 33 | *N* = 32 | *N* = 30 | *N* = 34 |  |  |  |  |  |  |  |  |
| 17 PSYRATS Negative Content | -.07 | .24 | .24 | -.29 | -.25 | .02 | -.03 | .09 | .11 | .11 | -.04 | -.04 | -.06 | -.01 | .24 | .71^***^ | ___ |  |  |  |  |  |  |
|  | *N* = 34 | *N* = 30 | *N* = 34 | *N* = 33 | *N* = 33 | *N* = 33 | *N* = 33 | *N* = 33 | *N* = 33 | *N* = 33 | *N* = 33 | *N* = 33 | *N* = 32 | *N* = 30 | *N* = 34 | *N* = 34 |  |  |  |  |  |  |  |
| 18 PSYRATS Loudness | -.25^a^ | .23^a^ | .17^a^ | .11 | .29^a^ | .32 | .04 | .27^a^ | .19^a^ | .15 | .21^a^ | .41^*^ | -.27^a^ | -.18^a^ | .53^a **^ | .59^***^ | .34^*^ | ___ |  |  |  |  |  |
|  | *N* = 34 | *N* = 30 | *N* = 34 | *N* = 33 | *N* = 33 | *N* = 33 | *N* = 33 | *N* = 33 | *N* = 33 | *N* = 33 | *N* = 33 | *N* = 33 | *N* = 32 | *N* = 30 | *N* = 34 | *N* = 34 | *N* = 34 |  |  |  |  |  |  |
| 19 BAVQ-R Persecutory Beliefs | -.30^a^ | .69^a ***^ | .45^a *^ | .17 | .07^a^ | .42 ^*^ | .21 | .14^a^ | .19^a^ | .30 | .24^a^ | .38^*^ | -.17^a^ | -.30^a^ | .17^a^ | .54^**^ | .64^***^ | .47^a **^ | ___ |  |  |  |  |
|  | *N* = 31 | *N* = 28 | *N* = 31 | *N* = 31 | *N* = 31 | *N* = 31 | *N* = 31 | *N* = 31 | *N* = 31 | *N* = 31 | *N* = 31 | *N* = 31 | *N* = 30 | *N* = 28 | *N* = 31 | *N* = 31 | *N* = 31 | *N* = 31 |  |  |  |  |  |
| 20 BAVQ-R Resistance | -.14^a^ | .43^a *^ | .58^a ***^ | -.31 | .04^a^ | .23 | -.03 | .15^a^ | .04^a^ | .26 | .13^a^ | .28 | -.03^a^ | -.11^a^ | .08^a^ | .40^*^ | .50^**^ | .24^a^ | .62^a***^ | ___ |  |  |  |
|  | *N* = 33 | *N* = 29 | *N* = 33 | *N* = 32 | *N* = 32 | *N* = 32 | *N* = 32 | *N* = 32 | *N* = 32 | *N* = 32 | *N* = 32 | *N* = 32 | *N* = 31 | *N* = 29 | *N* = 33 | *N* = 33 | *N* = 33 | *N* = 33 | *N* = 30 |  |  |  |  |
| 21 VAY Voice dominance | -.24 | .58 ^***^ | .40^*^ | .19 | .12 | .45^**^ | .21 | .02 | .21 | .24 | .17 | .33 | -.20 | -.22 | .11 | .47^**^ | .46^**^ | .27 | .80^***^ | .54^**^ | ___ |  |  |
|  | *N* = 34 | *N* = 30 | *N* = 34 | *N* = 33 | *N* = 33 | *N* = 33 | *N* = 33 | *N* = 33 | *N* = 33 | *N* = 33 | *N* = 33 | *N* = 33 | *N* = 32 | *N* = 30 | *N* = 34 | *N* = 34 | *N* = 34 | *N* = 34 | *N* = 31 | *N* = 33 |  |  |  |
| 22 VAY Voice intrusiveness | -.16^a^ | .55^a **^ | .40^a *^ | .08 | .14^a^ | .37^*^ | .06 | .15^a^ | .10^a^ | .30 | .27^a^ | .42^*^ | -.09^a^ | -.17^a^ | .62^a ***^ | .42^*^ | .39^*^ | .43^a *^ | .62 ^a ***^ | .47^a**^ | .46^**^ | ___ |  |
|  | *N* = 34 | *N* = 30 | *N* = 34 | *N* = 33 | *N* = 33 | *N* = 33 | *N* = 33 | *N* = 33 | *N* = 33 | *N* = 33 | *N* = 33 | *N* = 33 | *N* = 32 | *N* = 30 | *N* = 34 | *N* = 31 | *N* = 34 | *N* = 34 | *N* = 31 | *N* = 33 | *N* = 34 |  |  |
| 23 VAY Hearer distance | -.11^a^ | .30^a^ | .43^a *^ | -.12 | .18^a^ | .37^*^ | .05 | .17^a^ | .26^a^ | .18 | -.04^a^ | .24 | .00^a^ | -.01^a^ | -.06^a^ | .25 | .21 | .14^a^ | .49 ^a **^ | .71^a***^ | .69^***^ | .31  .26 | ___ |
|  | *N* = 34 | *N* = 30 | *N* = 34 | *N* = 33 | *N* = 33 | *N* = 33 | *N* = 33 | *N* = 33 | *N* = 33 | *N* = 33 | *N* = 33 | *N* = 33 | *N* = 32 | *N* = 30 | *N* = 34 | *N* = 34 | *N* = 34 | *N* = 34 | *N* = 31 | *N* = 33 | *N* = 34 | *N* = 34 |  |
| 24 VAY Hearer dependence | -.11 | .35 | .18 | .21 | .09 | -.17 | .05 | .13 | -.07 | .49^**^ | .56^**^ | .34 | -.45^*^ | -.53^**^ | .18 | -.17 | -.23 | .08 | .11 | -.11 | -.09 | .16 | -.28 |
|  | *N* = 32 | *N* = 28 | *N* = 32 | *N* = 31 | *N* = 31 | *N* = 31 | *N* = 31 | *N* = 31 | *N* = 31 | *N* = 31 | *N* = 33 | *N* = 31 | *N* = 30 | *N* = 28 | *N* = 32 | *N* = 32 | *N* = 32 | *N* = 32 | *N* = 33 | *N* = 31 | *N* = 32 | *N* = 32 | *N* = 32 |
| *Note.* BDI-II = Beck’s Depression Inventory - II; BAI = Beck Anxiety Inventory; PROQ-3 = shortened Person's Relating to Others Questionnaire; SCS = Social Comparison Scale; mSCS = Social Connectedness Scale; PSYRATS = Psychotic Symptom Rating Scales; BAVQ-R = Beliefs about Voices Questionnaire-Revised; VAY = The Voice and You. ^a^ Pearson’s *r* correlation coefficient (Bootstrapped *N* = 2000, BCa95%CI) reported instead of Spearman’s rho correlation coefficient as both correlation analysis variables were normally distributed.  * *p* < .05, ** *p* < .01, *** *p* < .001. | | | | | | | | | | | | | | | | | | | | | | | |
