# Supplementary material for: Distress factors of voice‐hearing in young people and social relating: Exploring a cognitive‐interpersonal voice‐hearing model
Source: Psychol Psychother. 2022 Jun 30;95(4):939–57. doi: 10.1111/papt.12411 (PMC9795969; doi:10.1111/papt.12411)
Supplement: Supplementary file 6 — Table S5 [file PAPT-95-939-s004.docx]

**Supplementary Material Table 5.** *Summary of the study hypotheses and hypothesis testing outcomes.*

| Hypothesis | Outcome | Decision |
| --- | --- | --- |
| **Relating to voices and voice-related distress** | |  |
| *Hypothesis 1.* Voice dominance and intrusiveness, hearer’s distance and resistance mode of responding will be related to voice-related distress. | Voice dominance, voice intrusiveness and a resistance mode of responding were significantly correlated with voice-related distress. Hearer’s distance was not related to voice-related distress. Persecutory beliefs were taken into account with or without depression and anxiety levels. Results showed that none of the hypothesised relationships were any longer significant. | Partially supported |
| *Hypothesis 2.* Hearer’s distancing from the voices and using a resistance mode of responding will be related to voice dominance. | Hearer’s distance and resistance was significantly correlated with voice dominance | Supported |
| **Beliefs about voices, phenomenological voice characteristics and voice-related distress** | |  |
| *Hypothesis 3.* Persecutory beliefs about the voices will be related to voice-related distress, independent from other voice characteristics such as their content, frequency, and loudness. | Persecutory beliefs were significantly related with voice-related distress when controlling for voice frequency. Controlling for the effect of loudness or negative voice content rendered the relationship between persecutory beliefs and voice-related distress statistically non-significant. | Partially supported |
| **Relating to voices and relating to others** | |  |
| *Hypothesis 4.* Relating from a position of distance to voices will be associated with distant relating styles with others (neutral distant and lower distant styles), while relating from a position of dependence to voices will be associated with relating dependently with social others (lower close lower neutral style and neutral close styles). | Neutral close relating (fear of separation and of being alone) was related to hearer distance. Neutral distant (suspicious, uncommunicative and self-reliant) and upper distant types of relating (sadistic, intimidating and tyrannising) were significantly associated to hearer dependence. | Rejected |
| *Hypothesis 5.* Relating from a position of dependence with the voices will be negatively associated with social connectedness and social belongingness. | Greater dependence relating styles toward the voices was related to lower perceived social belongingness and connectedness. | Supported |
